# Does sex matter? Longitudinal course and predictors of knowledge about immunosuppressant medication in patients after kidney transplantation: a KTx360° substudy

**DOI:** 10.3389/frtra.2026.1697923

**Published:** 2026-02-05

**Authors:** Mariel Nöhre, Deborah Meier, Julia Talamo, Uwe Tegtbur, Lars Pape, Mario Schiffer, Martina de Zwaan

**Affiliations:** 1 Department of Psychosomatic Medicine and Psychotherapy, Hannover Medical School, Hannover, Germany; 2 IGES Institute, Berlin, Germany; 3 Department of Rehabilitation and Sports Medicine, Hannover Medical School, Hannover, Germany; 4 Department of Pediatrics II, University Hospital of Essen, University of Duisburg-Essen, Essen, Germany; 5 Department of Nephrology and Hypertension, University Hospital Erlangen, Erlangen, Germany

**Keywords:** adherence, health literacy, immunosuppressive medication, kidney transplantation, renal transplantation

## Abstract

**Background:**

To ensure long term graft and patient survival after kidney transplantation, the correct intake of the immunosuppressive medication is mandatory. To correctly administer the medication, specific knowledge is required. While the significance of adherence has been recognized by many, the potential adverse effects of insufficient knowledge levels—often associated with poor health literacy—have been overlooked for a long time; therefore, little is known about sex-specific differences and other predictors of knowledge in this patient group.

**Methods:**

We analyzed the longitudinal course of the self-developed and previously successfully applied knowledge test in kidney transplant recipients participating in the KTx360° trial over a period of up to three years. The patients participated in a multidisciplinary aftercare program that included case management, psychosocial assessments and interventions, as well as exercise assessments and interventions, supported by telemedicine. We aimed to identify potential baseline predictors of knowledge trajectories, with a specific focus on sex-specific differences.

**Results:**

The analysis sample, which consisted of participants with at least one valid measurement on the knowledge test, included 783 adult patients (41.6% women) with a mean age of 52.3 years (SD 13.6). Knowledge levels improved significantly over the period of the KTx360° trial. Especially younger male participants and men not living in a partnership showed an increase in knowledge levels.

**Conclusions:**

Over the period of the KTx360° trial we observed an increase in knowledge, mainly in patients with below-average baseline knowledge levels. While some improvements might be due to the catch-up effect, other changes suggest a different response to the same stimulus. In sex-specific analyses, we found higher knowledge levels in female participants at the start, but sex did not impact the progression of knowledge levels. Since there was no control group, it is not possible to determine the program's effect on knowledge levels.

## 1 Introduction

Kidney transplantation is the treatment of choice for patients with end-stage renal disease (ESRD), as it is associated with superior quality of life (1). In Germany, the waiting time for a post-mortem donated kidney is on average 8–10 years, with waiting times exceeding 10 years becoming increasingly common (2). Securing patient and transplant survival is one of the main goals in post-transplant care.

One crucial aspect associated with reduced graft survival is suboptimal adherence to the immunosuppressive medication. As recent studies show, patients with suboptimal adherence have an increased risk for rejection episodes as well as graft loss (3, 4). Adherence is a complex construct that was defined by the WHO more than 20 years ago (5). A variety of factors are associated with and able to influence adherence behavior, one of which is knowledge regarding the correct intake of the medication and dealing with potential problems that might arise when taking this medicine. Knowledge can be understood as a part of health literacy. The WHO defines this concept as “cognitive and social skills which determine the motivation and ability of individuals to gain access to, understand, and use information in ways which promote and maintain good health” (6). In the literature, a weak association between health literacy and adherence has been described (7). The same has been found for knowledge levels as they may be directly associated with adherence behavior (8, 9). However, knowledge gaps may also lead to issues that are not defined as non-adherence, such as difficulties in managing the medication, for example, a lack of insight into potential interactions with other medications and nevertheless might lead to adverse outcomes (8, 9).

As part of the KTx360° trial (10, 11), we evaluated knowledge levels regarding immunosuppressive medications in a group of kidney transplant recipients at the baseline psychosocial evaluation (12). While only about 70% of the questions were answered correctly, a better knowledge level was associated with being in a partnership, better cognitive functioning, speaking German as the mother tongue, and being female. Little is known about the factors that have the potential to influence the course of knowledge levels, despite the importance of understanding them for developing and implementing training and strategies to improve knowledge levels, especially in patients with limited knowledge.

Based on this, we aimed to investigate the change in knowledge level, as measured by the knowledge test, over the duration of the KTx360° trial (10, 11), where we continued to ask patients to complete the knowledge test at every psychosocial evaluation, addressed the results of the tests and corrected false answers in every psychosocial contact that occurred with the patient. Additionally, we wanted to know if the differences in knowledge levels we detected at the first appointment with the patients also persisted over the course of the trial. In particular, we aimed to determine whether sex-based differences found at baseline, that are of increasing interest in KTx cohorts (13, 14), remained throughout the course of KTx360°. Apart from that, we evaluated whether the variables associated with the scores on the knowledge test at baseline also influenced and shaped the course of the knowledge level.

## 2 Methods

### 2.1 Sample selection

This study represents a *post hoc* analysis of data from the KTx360° trial. The participants were included within the structured post-transplant care program KTx360° in the transplant centers of Hannover Medical School and Hann. Münden in Lower Saxony, Germany (10, 11). Details about the KTx360° program are described elsewhere (10, 11). Between May 2017 and September 2020 data were collected within this trial. The participants underwent a psychosocial assessment twice to four times a year, conducted by a mental health professional (physician or psychologist), which included a knowledge assessment as described below. Additionally, the participants were asked to complete several questionnaires. Participants of the KTx360° trial with an inability to speak, read, or understand the German language, with visual impairment or a known history of severe developmental delay, hindering them from completing the knowledge test, were excluded from this sub-study. In total, 783 participants were included in the analysis who completed the knowledge test at least once. The Institutional Ethics Review Board of Hannover Medical School approved the study (Number 3464–2017). All participants gave written informed consent.

### 2.2 Knowledge test

Patients were asked to complete a self-developed questionnaire comprising eight multiple-choice questions concerning necessary and more specific information regarding the immunosuppressive medication and its usage. The instrument is expert-based and has previously been used in a different smaller sample of patients after KTx at Hannover Medical School (15). The questions of the knowledge test are considered essential by the transplant nephrologists who designed it. The correct handling of immunosuppressive medication, including the aspects covered in the knowledge test, is explained to patients in the days following KTx and during their visits to the transplant outpatient clinic. Additionally, the baseline values and correlates of a subgroup of patients in the KTx360° study (*N* = 702) were reported earlier (12).

The questionnaire was designed to detect lack of knowledge regarding the immunosuppressive medication. As the questions aim at basic knowledge, the patients were expected to answer all questions correctly.

The scoring is described in detail elsewhere (12). In short, some questions had more than one correct answer, and patients were encouraged to make multiple selections. Questions were judged to be answered correctly overall if patients chose at least one correct answer without choosing a wrong answer at the same time. If the patient did not select an answer, the question was considered incorrectly answered. This scoring aligns with that of Bertram et al. (15) and de Boer et al. (12). The total number of correctly answered questions was calculated and used for further analyses.

Patients were asked to complete the knowledge tests at each psychosocial assessment, which took place four times during the first year post-transplantation and up to two times in subsequent years (10, 11). The knowledge test was discussed with the patient, and wrong answers were corrected by the medical professional completing the psychosocial assessment.

### 2.3 Hospital anxiety and depression scale (HADS)

The German version of the Hospital Anxiety and Depression Scale (HADS) is a self-report instrument, which was used to measure levels of anxiety and depression (16–18). It is specifically designed to evaluate levels of anxiety and depression in patients with physical illnesses. The questionnaire consists of two subscales, “depression” and “anxiety,” with seven items each. The items are rated between 0 and 3, leading to a sum score between 0 and 21, with higher results indicating higher levels of depression or anxiety. At baseline, Cronbach's *α* in our sample was 0.86 for depression and 0.82 for anxiety (*n* = 755).

### 2.4 Perceived social support (F-SozU K7)

Perceived social support was assessed using the German F-SozU K7 at enrollment (19, 20). The instrument includes seven items rated on a 5-point Likert scale, from 1 (“does not apply”) to 5 (“exactly applicable”). Therefore, total scores range from 7 to 35, with higher scores indicating greater perceived social support. At baseline, Cronbach's *α* in our sample was 0.90 (*n* = 745).

### 2.5 Medical, sociodemographic, and transplant-specific variables

Sociodemographic and donation-specific variables, including sex, age at enrollment, partnership status, years of education, first language, type of donation (living or deceased donor), and time since KTx at enrollment, were collected using a self-report questionnaire. Missing information was taken from the medical records. The estimated glomerular filtration rate (eGFR) was extracted from the medical records at the time of enrollment into the study.

### 2.6 Data analysis

For each variable descriptive statistics (percentages, means and standard deviations, medians, minimum and maximum) were calculated. We used independent samples *t*-tests to compare continuous data between female and male participants. Chi-square tests were used for categorical data.

Knowledge trajectories were analyzed in KTx360° participants using a longitudinal mixed model with the knowledge sum score as the dependent variable (individuals at level two and their measurement points at level one). The average change over time in knowledge scores within the KTx360° cohort was estimated by the fixed effect of the predictor months in KTx360°. Level two covariates were sex, age, years of education, partnership status, first language, donor type, level of depression (HADS), time since KTx at enrollment. The mixed models include a random intercept and random slope for months in KTx360° allowing for variation in intercept and slope between individuals.

To examine the association of the above-mentioned level two covariates and knowledge level over time, we conducted separate analyses predicting knowledge level by the interaction of time in KTx360° and each covariate. Accordingly, we performed eight mixed models, each with an interaction term for the respective covariate and time in KTx360°. Additionally, we performed separate analyses for all seven covariates for both men and women. To account for multiple testing across the eight related models, *p*-values were adjusted using the Benjamini–Hochberg procedure (false discovery rate, FDR). The statistical analyses were performed using R [version 4.4.1, (21)] and the nlme (22) and lme4 (23) packages.

## 3 Results

### 3.1 Sample characteristics

For this analysis, we included adult participants treated at MHH and Hann. Münden resulting in a sample of 783 participants who completed the knowledge test at least once, with an average of 3.68 measurement points (SD = 1.53, Min = 1, Max = 7) over a period of 11.13 months (SD = 9.55, Min = 0, Max = 39). Due to missing data on the 8 covariates, the sample size in the regression analyses was reduced to *N* = 725.

Descriptives of the sample with valid information of at least one knowledge test (*n* = 783), and the sample with complete data on all covariates that was included in the multilevel regression analyses (*n* = 725) are reported in Table 1. Additionally, we reported results for female and male participants within the sample with complete data for regression analyses separately. Results are also depicted in Table 1. Female participants were less likely in a partnership, reported less years of education and had higher scores in the HADS anxiety score at baseline.

**Table 1:** Baseline characteristics.

Variables	Sample with at least one knowledge test *N* = 783	Sample with complete data for regression analyses			
		Total sample *N* = 725	Females *N* = 299	Males *N* = 426	Statistics
Sex, *n* (%) female	326 (41.6%)	299 (41.2%)			
Age, years					t (658.3) = −0.40, *p* =.688
Mean (SD)	52.3 (13.6)	52.2 (13.7)	52.0 (13.3)	52.4 (13.9)	
Median (min, max)	54.0 (18.0, 81.0)	54.0 (18.0, 81.0)	55.0 (19.0, 81.0)	53.5 (18.0, 81.0)	
Time since KTx at enrollment, years					t (710.9) = −1.37, *p* = .171
Mean (SD)	5.6 (5.7)	5.7 (5.8)	5.5 (5.6)	5.8 (5.9)	
Median (min, max)	4.0 (1.0, 34.0)	4.0 (1.0, 34.0)	4.0 (1.0, 33.0)	4.0 (1.0, 34.0)	
Donor type, *n* (%)	*n* = 782				*χ*² (1) = 2.00, *p* = .157
Living donation	224 (28.6%)	211 (29.1%)	78 (26.1%)	133 (31.2%)	
Partnership, *n* (%)	*n* = 754				**χ ² (1) = 4.20, *p*** = **.041**
Yes	525 (69.6%)	509 (70.2%)	197 (65.9%)	312 (73.2%)	
Native language, *n* (%)	*n* = 740				χ ² (1) = 0.06, *p* = .807
Other than German	75 (10.1%)	74 (10.2%)	32 (10.7%)	42 (9.9%)	
Years of education, years	*n* = 753				**t (661.3) = −2.31, *p*** = **.021**
Mean (SD)	12.3 (2.5)	12.3 (2.45)	12.1 (2.4)	12.5 (2.5)	
Median (min, max)	12.0 (8.0, 18.0)	12.0 (8.0, 18.0)	12.0 (8.0, 18.0)	12.0 (8.0, 18.0)	
eGFR, value	*n* = 750	*n* = 717	*n* = 295	*n* = 422	t (648.48) = −1.22, *p* = .224
Mean (SD)	45.7 (18.3)	45.6 (18.0)	44.6 (17.6)	46.3 (18.3)	
Median (min, max)	43.3 (10.3, 123.3)	43.1 (10.4, 123.3)	42.9 (10.4, 104)	43.2 (11.5, 123)	
HADS, anxiety score	*n* = 755				**t (620.22) = 2.28, *p*** = **.023**
Mean (SD)	5.1 (3.9)	5.1 (3.9)	5.48 (4.00)	4.8 (3.8)	
Median (min, max)	4.0 (0.0, 19.0)	4.0 (0.0, 19.0)	5.00 (0, 17.5)	4.00 (0, 19.0)	
HADS depression score	*n* = 755				t (680.62) = 0.72, *p* = .472
Mean (SD)	4.3 (3.9)	4.3 (3.9)	4.4 (3.7)	4.2 (4.1)	
Median (min, max)	3.0 (0.0, 21.0)	3.0 (0.0, 21.0)	4.0 (0.0, 19.0)	3.0 (0.0, 21.0)	
FSoZu	*n* = 745	*n* = 715	*n* = 294	*n* = 421	t (592.7) = −0.49, *p* = .624
Mean (SD)	30.0 (6.1)	30.1 (6.0)	29.9 (6.37)	30.2 (5.8)	
Median (min, max)	32.0 (7.0, 35.0)	32.0 (7.0, 35.0)	33.0 (7.0, 35.0)	32.0 (7.0, 35.0)	
Knowledge test at baseline, number of correctly answered questions	*n* = 761	*n* = 715	*n* = 296	*n* = 419	**t (604.35) = 2.28, *p*** = **.023**
Mean (SD)	5.69 (1.46)	5.71 (1.5)	5.9 (1.5)	5.6 (1.4)	
Median (min, max)	6.0 (1.0, 8.0)	6.0 (1.0, 8.0)	6.0 (1.0, 8.0)	6.0 (1.0, 8.0)	
100% correct answers in the Knowledge test at baseline, *n* (%)	*n* = 761 80 (10.5%)	*n* = 715 76 (10.6%)	*n* = 296 43 (14.5%)	*n* = 419 33 (7.9%)	**χ ² (1) = 7.39, *p*** = **.007**

eGFR, estimated glomerular filtration rate; F-SozU K7, perceived social support scale; HADS, Hospital anxiety and depression scale. We used independent samples *t*-tests to compare continuous data between female and male participants. Chi-square tests were used for categorical data. Bold *P*-values indicate significant results.

### 3.2 Knowledge trajectories

**Figure 1:**
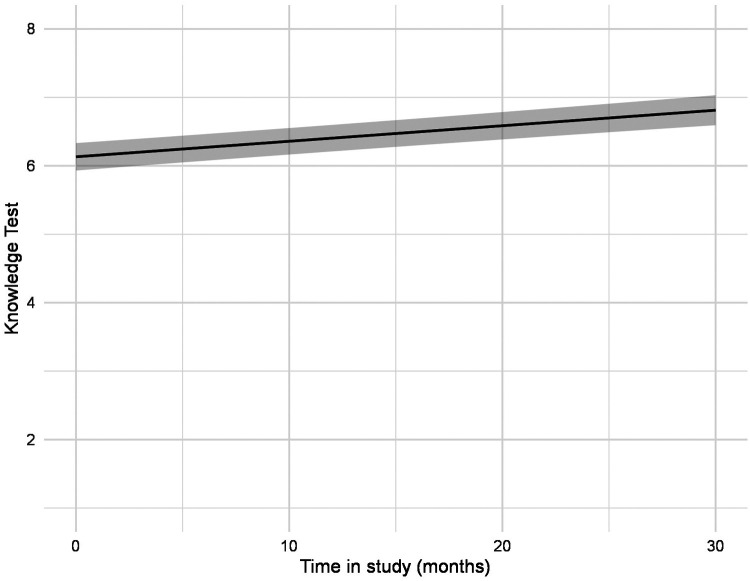
Estimated values of the knowledge test over time, adjusted for all covariates.

**Figure 2:**
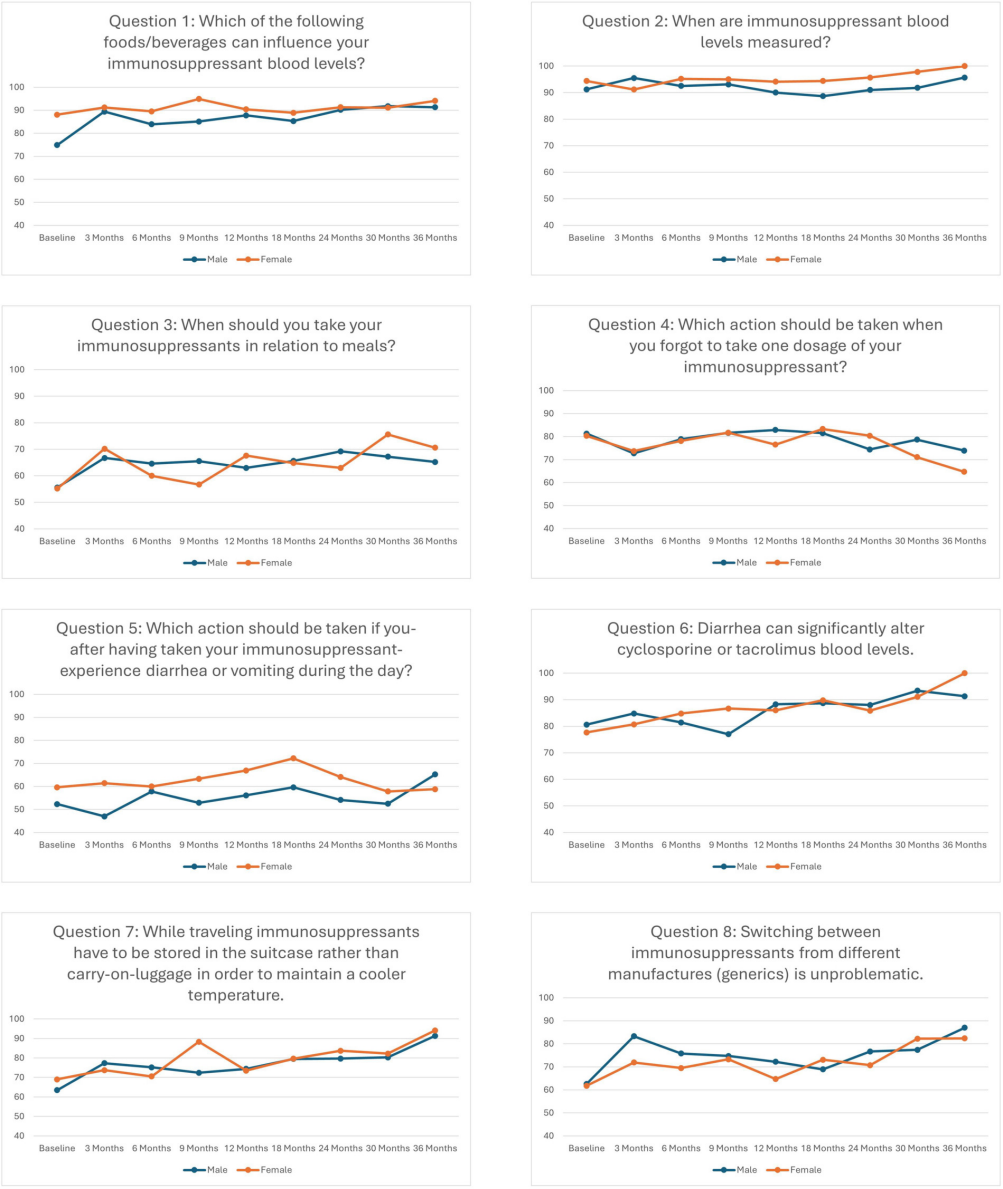
Percentage of female and male patients answering the separate items of the knowledge test correctly.

Overall, the number of correctly answered questions of the knowledge test increased significantly over time (Figure 1, Table 2). The mean percentage of correctly answered questions increased from 71.5% up to 83.4% (Figure 1, Table 3). At each assessment point between 10.5 and 28.2% of the participants answered all questions correctly (Table 3). Percentage of female and male patients answering the separate items of the knowledge test correctly are reported as well (Figure 2) and Supplementary Table 8). The proportion of participants who have answered a question correctly varies considerably between questions and time points, so that between 47.0% and 100.0% answered a question correctly. In order not to undermine the chosen evaluation method, we have refrained from making statistical comparisons between female and male participants at different time points.

**Table 2:** Time in study predicts the course of the knowledge level.

Predictors [category]	Estimates	CI	*p*
Intercept	6.10	5.90–6.30	**<0** **.** **001**
Time in KTx360°	0.02	0.02–0.03	**<0**.**001**
First language [non-German]	−0.73	−0.99 to −0.46	**<0**.**001**
Sex [male]	−0.24	−0.40 to −0.07	**0**.**005**
Partnership [no]	−0.24	−0.43 to −0.05	**0**.**014**
Donor type [postmortal]	−0.12	−0.31–0.06	0.197
Time since KTx at enrollment into study	−0.02	−0.03 to −0.00	**0**.**021**
Years of education	0.05	0.01–0.08	**0**.**008**
Age	−0.01	−0.02 to −0.00	**0**.**002**
Depression (HADS)	−0.03	−0.05 to −0.01	**0**.**012**
Random effects			
*σ* ^2^	1.16		
*τ*_00__Code_eFA_	0.73		
ICC	0.39		
N _Code_eFA_	725		
Observations	2125		

HADS, hospital anxiety and depression scale. reference categories: sex = female, first language = German, partnershi*p* = yes, donor type = living donation. Bold *P*-values indicate significant results (without Bonferroni correction).

**Table 3:** Knowledge test results over time: mean correct answers and percentage of participants with 100% correct answers.

Time in study	Sample size (*n*)	Mean (SD) correct answers	Percentage of correct answers	Percentage of 100% correct answers
Baseline	761	5.72 (1.44)	71.5	10.51
3 months	123	6.15 (1.36)	76.88	16.26
6 months	265	6.09 (1.47)	76.13	16.98
9 months	146	6.17 (1.45)	77.13	21.92
12 months	315	6.17 (1.39)	77.13	18.41
18 months	257	6.3 (1.35)	78.75	20.62
24 months	225	6.28 (1.4)	78.5	20.00
30 months	106	6.41 (1.23)	80.13	17.92
36 months	39	6.67 (1.18)	83.38	28.21

**Figure 3:**
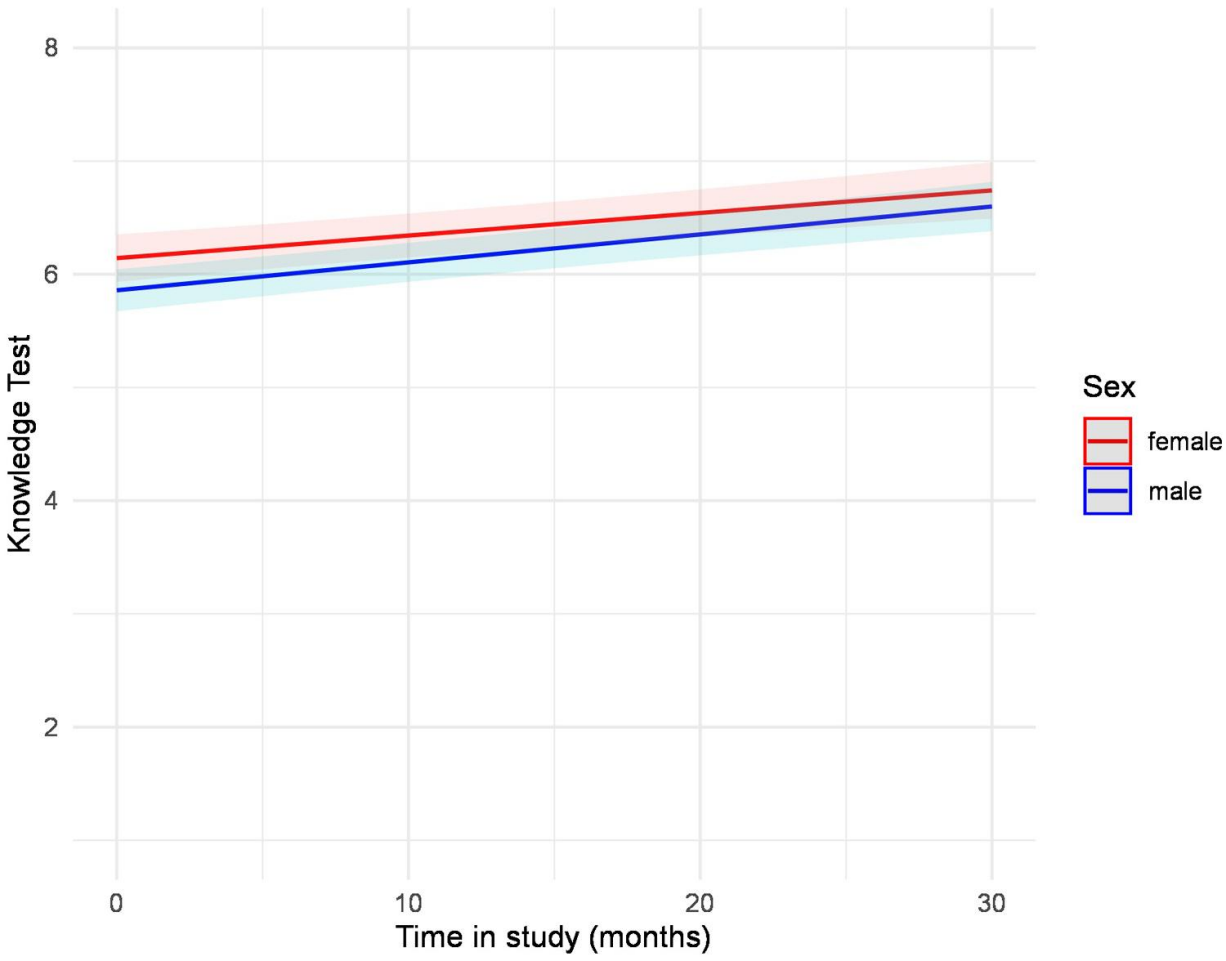
Estimated values of the interaction plot between the knowledge test over time and sex, adjusted for all covariates. The regression analysis is depicted in Supplementary Table 1.

**Figure 4:**
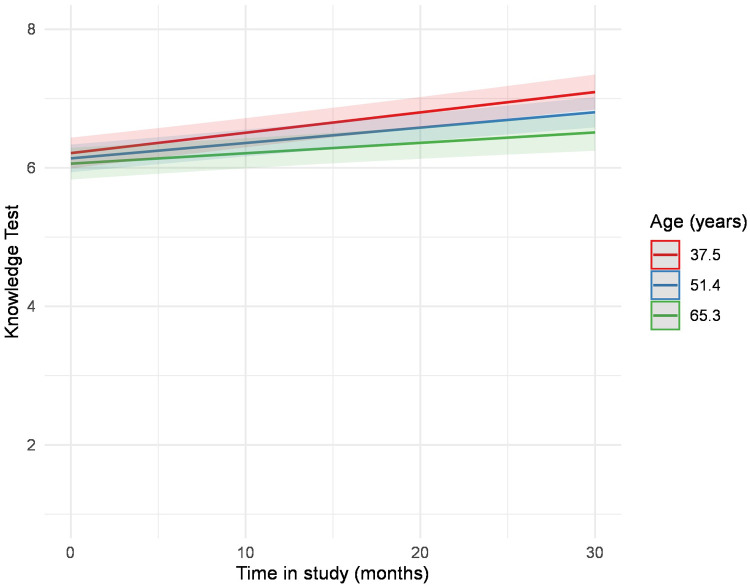
Estimated values of the interaction plot between the knowledge test over time and age, adjusted for all covariates. The regression analysis is depicted in Supplementary Table 2.

**Figure 5:**
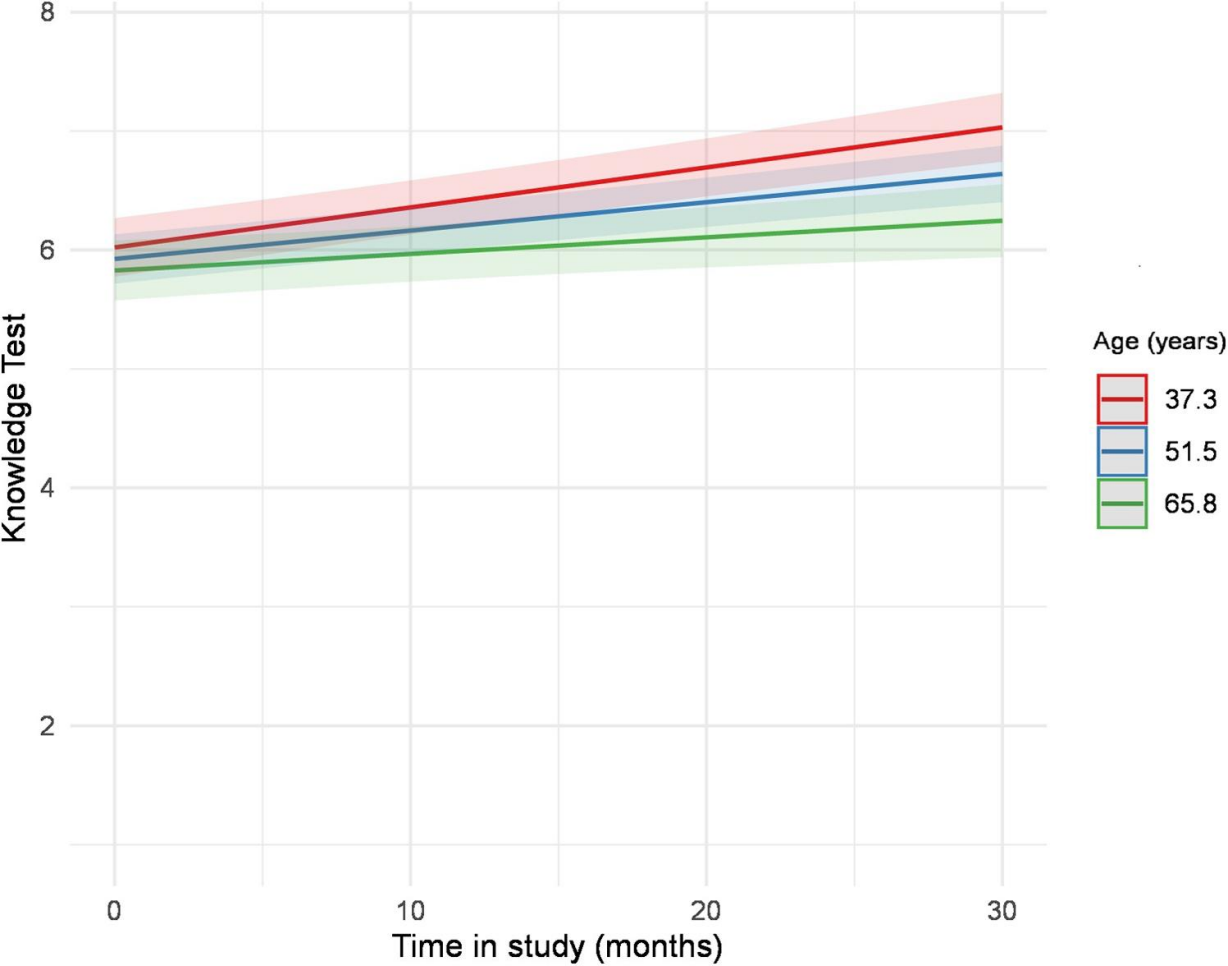
Estimated values of the interaction plot between the knowledge test over time and age, adjusted for all covariates in the male subsample. The regression analysis is depicted in Supplementary Table 4.

At baseline, female participants reached significantly higher knowledge scores (Table 1). The knowledge test trajectories did not differ between men and women (b = 0.00, *p* = .372). In both groups, as depicted in Figure 3, knowledge improved in a nearly parallel course, with women exhibiting better results at baseline and maintaining them over time (Table 4, Supplementary Table 1, Figure 3). We performed multiple mixed-model regression analyses to predict the knowledge test trajectories over time. Results from the individual models are presented with unadjusted *p*-values. After correction for multiple testing using the Benjamini–Hochberg procedure (FDR), all significant effects remained unchanged (Table 5). Results for age revealed a significant interaction term (b = −0.00, *p* = .006), indicating that younger participants showed a steeper increase in knowledge test results compared to older participants. (Table 5, Supplementary Table 2). This result is also shown in Figure 4, which displays the modelled trajectories for three age groups. Regarding the sex-segregated analyses, the interaction term for age was only significant for male participants (b = −0.00, *p* = .004) and not for female participants (b = −0.00, *p* = .381) (Table 5, Supplementary Tables 3, 4, Figure 5).

**Table 4:** Knowledge test results over time: mean number of correct answers separated by sex.

Time in study	Sample size (*n*) and mean (SD) number of correct answers (0–8)	
	Female	Male
Baseline	*n* = 318	*n* = 443
	5.86 (1.5)	5.62 (1.39)
3 months	*n* = 57	*n* = 66
	6.14 (1.3)	6.17 (1.42)
6 months	*n* = 104	*n* = 161
	6.09 (1.57)	6.1 (1.41)
9 months	*n* = 59	*n* = 87
	6.39 (1.29)	6.02 (1.55)
12 months	*n* = 135	*n* = 180
	6.2 (1.3)	6.14 (1.46)
18 months	*n* = 107	*n* = 150
	6.48 (1.25)	6.17 (1.4)
24 months	*n* = 92	*n* = 133
	6.35 (1.42)	6.23 (1.4)
30 months	*n* = 45	*n* = 61
	6.49 (1.36)	6.34 (1.14)
36 months	*n* = 16	*n* = 23
	6.75 (1.06)	6.61 (1.27)

**Table 5:** Regression results adjusted for all covariates on the interaction effects of time in study and each of the 8 covariates reported for the total sample and separately by sex.

Interaction term	Sample	Estimates	CI	*p*-value	p FDR
Sex * Time in study		0.00	−0.01–0.02	.372	.663
Age * Time in study	Total sample	−0.00	−0.00 to −0.00	.**006**	.**044**
	Female	−0.00	−0.00 to −0.00	.381	.663
	Male	−0.00	−0.00 to −0.00	.**004**	.**044**
Partnership * Time in study	Total sample	0.01	−0.00–0.02	.072	.264
	Female	−0.01	−0.02–0.01	.526	.723
	Male	0.02	0.01–0.04	.**002**	.**044**
Years of education * Time in study	Total sample	−0.00	−0.00–0.00	.708	.865
	Female	−0.00	−0.00–0.00	.593	.767
	Male	−0.00	−0.00–0.00	.309	.663
Depression (HADS) * Time in study	Total sample	−0.00	−0.00–0.00	.850	.890
	Female	−0.00	−0.00–0.00	.243	.663
	Male	−0.00	−0.00–0.00	.437	.687
Native language * Time in study	Total sample	0.02	−0.00–0.03	.055	.264
	Female	0.02	−0.00–0.05	.064	.264
	Male	0.01	−0.01–0.03	.378	.663
Time since KTx at enrollment into study * Time in study	Total sample	−0.00	−0.00–0.00	.292	.663
	Female	−0.00	−0.00–0.00	.392	.663
	Male	−0.00	−0.00–0.00	.486	.713
Donor type * Time in study	Total sample	−0.00	−0.01–0.01	.908	.908
	Female	0.00	−0.02–0.02	.840	.890
	Male	−0.00	−0.02–0.01	.841	.890

HADS, hospital anxiety and depression scale. To account for multiple testing across the eight related models, *p*-values were adjusted using the Benjamini–Hochberg procedure (false discovery rate, FDR). Bold *P*-values indicate significant results (without Bonferroni correction).

**Figure 6:**
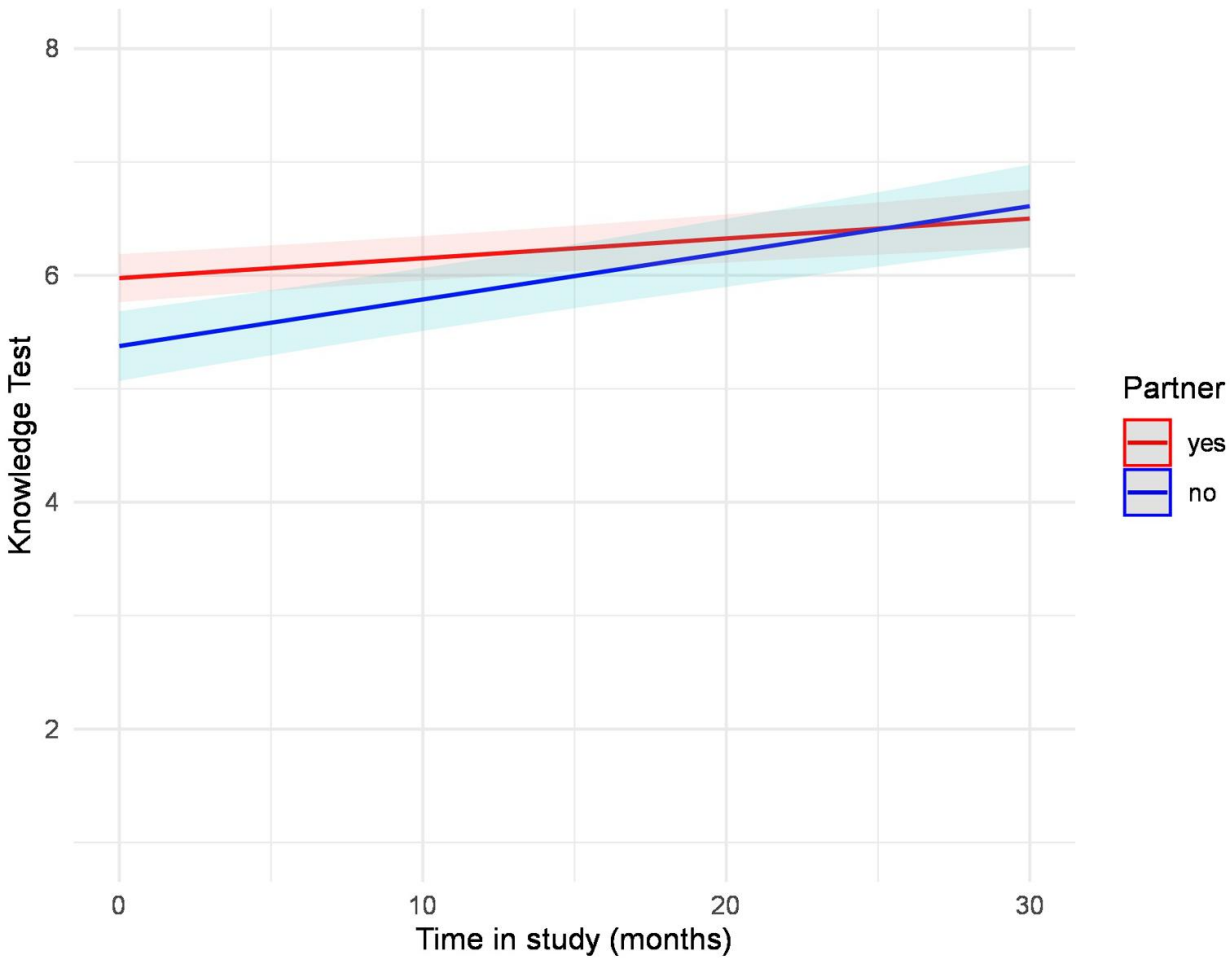
Estimated values of the interaction plot between the knowledge test over time and partnership, adjusted for all covariates in the male subsample. The regression analysis is depicted in Supplementary Table 7.

With respect to partnership, we found a significant interaction term only for male participants (b = .02, *p* = .002) but not for female participants (b = −0.01, *p* = .526) or the complete sample (b = 0.01, *p* = .072) (Table 5, Supplementary Table 5–S7). Male participants without a partner exhibited a higher increase in knowledge over time even reaching and exceeding the knowledge level of men with a partner (Figure 6).

No associations were found with educational level, level of depression, native language, time since KTx at enrollment into the study, or donor type (Table 5, Supplementary Tables 9–S23).

## 4 Discussion

Knowledge measured with the knowledge test increased over the course of the KTx360° trials, even though the knowledge levels that were reached still had room for improvement. With results of about 80% of the questions answered correctly, they appear to be in line with or even superior to other studies, even though the comparison is limited by the use of different instruments to measure knowledge (9, 24). Interestingly, only some of the variables we investigated were associated with the course of knowledge levels over time. As there is minimal research on this phenomenon so far, we would like to discuss our findings in detail.

Regarding sex, we saw a difference at baseline that is described in our previous manuscript, with women reporting better results on the knowledge test compared to men. Interestingly, there was a near-parallel development of the knowledge level, showing that sex does not influence the course of the knowledge test over time. Regarding the sex-based differences at baseline, our results are in line with the literature. Even though studies focusing on knowledge levels are scarce, this phenomenon has been found in other trials, including KTx recipients (9), patients with atrial fibrillation (25), and patients with cardiovascular diseases, including hypertension (26–28). However, only a few study groups have attempted to explain it. A possible explanation might be that women are more interested in the consequences of their disease and their health in general (25). As this might increase the susceptibility to educational campaigns, it could lead to better knowledge in women (25). In line with this argumentation strategy, Katz-Greenberg et al. (14) suggest a graft survival advantage due to better adherence to therapeutic recommendations and medication intake despite the more complex immunological situation for female transplant recipients. The authors emphasize that this hypothesis originates from sociocultural differences between men and women, which suggest that women show more awareness regarding health care-related topics and are more adherent to the recommendations of health care providers (14). These findings have to be understood in the context of the sex gap in KTx regarding access to the waiting list, access to transplantation, and transplantation outcomes (13, 14): Women are less frequently waitlisted, have a longer waiting time for a suitable transplant, and experience a higher rate of graft injury due to immunological factors. The reasons for this are multifaceted and complex and are not yet fully understood, even though this phenomenon is known among clinicians working in the field. Nate et al. proposed different potential explanations including the role of women as caregivers, stereotyping and stigma, social disadvantage and vulnerability (29). Based on this, one hypothesis might be that the stakes for women with end-stage kidney disease are particularly high to get waitlisted for a KTx, and that they require above-average knowledge compared to male patients. However, further research in this field is urgently needed to better understand the problem and develop potential solutions.

Surprisingly, only a few of the measured variables had an impact on the course of knowledge levels. Younger participants (specifically men) improved the most over time. The same could be found for men not living in a partnership. However, none of the variables we measured influenced the course of knowledge development in the female study population; in the total sample, only age showed a significant influence.

Concerning age, no differences at baseline could be detected, and younger participants improved more over time compared to older ones. This finding is in line with the literature, as the associations between higher age and lower knowledge levels, as well as suboptimal health literacy, have been observed before (30). Older age has been described as an independent risk factor for adverse drug events, with health literacy and knowledge about the medication as underlying factors (31–33). As health literacy can be understood as a necessary skill to acquire knowledge, this circumstance might explain why younger participants experience a stronger increase in the knowledge level compared to older ones.

In regard to partnership status, we found that not being in a partnership is associated with lower knowledge levels at baseline, which is in line with our own previous findings and other research as well (12, 15, 34). Others have described an association between being single and lower levels of health literacy in KTx patients (35, 36). It can be hypothesized that the partner might fulfill different functions, like accompanying the KTx patient to the doctor's appointment (12), assimilating and digesting health-related information (37), influencing medical decisions (38), and providing information on health-related topics (39). This phenomenon has been described as distributed literacy before (40); however, as Chisholm-Burns et al. (30) postulate, there is still more research needed on the effects of the health literacy levels of caregivers, who are often also partners or spouses, on long-term outcomes in organ transplantation. Interestingly, the knowledge level of male participants not living in a partnership improved during the KTx360° trial, even exceeding the knowledge level of patients living in a partnership at some point. We observed a similar development in younger male patients with a longer time after transplantation with regard to adherence (41).

Some aspects require further consideration. As this was a *post hoc* analysis, the findings should be interpreted with caution. The number of observations decreased over time mainly because participants entered the study at different time points. Multilevel modeling accounted for this unbalanced data structure, although some selective nonresponse cannot be entirely ruled out. Due to the design of our trial lacking a control group (11, 41), it remains unclear whether the improvement of knowledge is a direct result of the KTx360° trial. However, knowledge levels are independent of time since transplantation (12), and a natural increase in knowledge level is not to be expected. Interventions can improve knowledge levels and health literacy, as has been shown by others (42, 43). At the same time, as described by the Hawthorne effect, the observation itself might positively influence the outcome, in this case knowledge levels (44, 45). Additionally, an increase of correctly answered questions over time can be attributed to familiarity with the knowledge test as well as gain of knowledge. The methodology applied in this sub-study makes it impossible to differentiate between these potential explanations based on statistical analyses. As mistakes in the knowledge test were discussed with and explained to the participants, we are optimistic that at least some of the improvement in the knowledge test results over time is attributable to a gain in knowledge. To examine this aspect in future studies, a qualitative assessment or the use of slightly altered questions over the course of time would be necessary to evaluate the capacities for knowledge transfer.

It is necessary to point out that participants of the KTx360° trial with an inability to speak, read, or understand the German language, with visual impairment or a known history of severe developmental delay, hindering them from completing the knowledge test, were excluded from this sub-study. Specifically, patients meeting these criteria might experience difficulties receiving information and addressing ambiguities in clinical routine, which could lead to below-average knowledge levels in this patient group. Therefore, this sub-study might underestimate the knowledge gap. However, it is important to note that although patients meeting the criteria were not included in this sub-study, they did participate in the KTx360° trial. They were able to take advantage of the services offered and were often supported by caregivers, so that clinical care was not compromised. Nevertheless, in terms of equity, also in the context of being waitlisted for KTx there is a chance that these groups might have a disadvantage, especially patients with a language barrier. While a recent meta-analysis comprising data from five European countries found no association between migration status, which can be associated with language barriers, and being waitlisted for KTx (46), data from Germany on this phenomenon are currently unavailable and further research is required. A recent meta-analysis by Godoi et al. (47) underlines that educational interventions are able to improve disparities in access to KTx. Specifically, “home-based” approaches were successful in improving access to KTx. Therefore, regarding equity in access to KTx, educational interventions at an earlier stage seem to be an effective tool.

Apart from that, it is important to note that the knowledge test we used did not meet psychometric criteria (12). As others have shown, tests that align with psychometric criteria are rare (8). At the time of trial planning, since no validated German instrument was available, we decided to use the questionnaire that had already been successfully administered before (15). There are no indications of sex-specific response patterns when looking at the item level.

We are unaware of the long-term trajectories of knowledge levels after completion of the KTx360° trial. Additionally, we do not know the effect of knowledge level trajectories on clinical outcomes, including graft and patient survival. We refrained from investigating the association between knowledge level and adherence in this sub-study. As described by Zhang et al. (7), merely a weak association was often detected between knowledge levels and adherence, as well as between health literacy and adherence. This might be because adequate knowledge provides a basic framework for appropriate behavior in the therapeutic context, which is usually not included in instruments that measure adherence. Therefore, knowledge gaps in even basic aspects are often not recognized in routine clinical care. Due to the complex nature of the relationship between these variables, as described in the introduction section, a different study design would be needed to do it justice including more specific instruments.

Interestingly, of the variables we investigated, only very few influenced knowledge levels over time. It is important to note that patient-related factors often intersect and need to be understood in a broader context that influences health literacy as well as knowledge levels. This means, as Chisholm-Burns (30) described it well, that a variable like old age may serve as a proxy for, e.g., cognitive impairment. In our trial, we evaluated a limited number of variables lacking important variables like the socioeconomic status; however, for future studies, a more intense focus on potential intersections and a broader range of variables should be included to get a better understanding.

In conclusion, over the course of the KTx360° trial we observed an increase in knowledge level predominantly in patients with below average knowledge levels at baseline. While some improvements might be explained by the “catch-up effect”, e.g., in male patients not living in a partnership, other developments suggest a different response to the same stimulus, e.g., younger patients showed a higher increase in knowledge levels compared to older ones. With regard to sex-specific analyses, we identified higher knowledge levels in female participants at baseline, while sex did not influence the course of knowledge levels. As there was no control group included, it is difficult to comment on the effectiveness of the program. Nevertheless, only about 80% of the knowledge test, which comprised only questions considered essential by transplant nephrologists, were answered correctly. Due to the potential risks caused by the inappropriate handling of immunosuppressive medication, the current knowledge level should not be considered sufficient, and the inclusion of knowledge transfer into routine clinical care seems mandatory as well as the reinforcement of health literacy. Other tools like leaflets or online programs might be helpful additions; however, it is important to keep in mind that educational materials must be written in line with the average patient's literacy level (34). To address the needs of patients with a language barrier, information in other languages should be made available. For patients who require a caregiver's assistance, specific effort should be made to adequately inform the caregiver. From an equity perspective, educational interventions should already be implemented in patients with ESRD to enable access to the transplant waitlist in disadvantaged groups (47). Regarding future studies, different variables should be included, as the ones we investigated have contributed little to the understanding of the longitudinal development of knowledge levels. Additionally, impact of knowledge levels on long-term outcomes needs to be studied in prospective longitudinal trials.

## Data Availability

The raw data supporting the conclusions of this article will be made available by the authors, without undue reservation.
