# Supplementary material for: Does sex matter? Longitudinal course and predictors of knowledge about immunosuppressant medication in patients after kidney transplantation: a KTx360° substudy
Source: Front Transplant. 2026 Feb 5;5:1697923. doi: 10.3389/frtra.2026.1697923 (PMC12916695; doi:10.3389/frtra.2026.1697923)
Supplement: Supplementary file 1 [file Datasheet1.docx]

Supplementary Material

# Supplementary Tables

# Supplementary Table 1. Sex does not predict the course of the knowledge level: total sample

# Supplementary Table 2. Age predicts the course of the knowledge level: total sample

# Supplementary Table 3. Age does not predict the course of the knowledge level: female subsample

# Supplementary Table 4. Age predicts the course of the knowledge level: male subsample

# Supplementary Table 5. Partnership does not predict the course of the knowledge level: total sample

# Supplementary Table 6. Partnership does not predict the course of the knowledge level: female subsample

# Supplementary Table 7. Partnership predicts the course of the knowledge level: male subsample

Supplementary Table 8: Percentage of female and male patients answering the separate items of the knowledge test correctly.

Supplementary Table 9: Years of education do not predict the course of the knowledge level: total sample

Supplementary Table 10: Years of education do not predict the course of the knowledge level: female subsample

Supplementary Table 11: Years of education do not predict the course of the knowledge level: male subsample

Supplementary Table 12: Depression (HADS-D) does not predict the course of the knowledge level: total sample

Supplementary Table 13: Depression (HADS-D) does not predict the course of the knowledge level: female subsample

Supplementary Table 14: Depression (HADS-D) does not predict the course of the knowledge level: male subsample

Supplementary Table 15: First language does not predict the course of the knowledge level: total sample

Supplementary Table 16: First language does not predict the course of the knowledge level: female subsample

Supplementary Table 17: First language does not predict the course of the knowledge level: male subsample

Supplementary Table 18: Time since KTx at enrollment into study does not predict the course of the knowledge level: total sample

Supplementary Table 19: Time since KTx at enrollment into study does not predict the course of the knowledge level: female subsample

Supplementary Table 20: Time since KTx at enrollment into study does not predict the course of the knowledge level: male subsample

Supplementary Table 21: Donor type does not predict the course of the knowledge level: total sample

Supplementary Table 22: Donor type does not predict the course of the knowledge level: female subsample

Supplementary Table 23: Donor type does not predict the course of the knowledge level: male subsample

Supplementary Table 1. Sex does not predict the course of the knowledge level: total sample

| Predictors [category] | Estimates | CI | p |
| --- | --- | --- | --- |
| Intercept | 6.16 | 5.95 – 6.37 | **<0.001** |
| Sex [male] | -0.28 | -0.48 – -0.09 | **0.004** |
| Time in KTx360° | 0.02 | 0.01 – 0.03 | **<0.001** |
| First language [non-German] | -0.73 | -0.99 – -0.46 | **<0.001** |
| Partnership [no] | -0.24 | -0.43 – -0.05 | **0.014** |
| Donor type [postmortal] | -0.12 | -0.31 – 0.06 | 0.196 |
| Time since KTx at enrollment into study | -0.02 | -0.03 – -0.00 | **0.021** |
| Years of education | 0.05 | 0.01 – 0.08 | **0.009** |
| Age | -0.01 | -0.02 – -0.00 | **0.002** |
| Depression (HADS) | -0.03 | -0.05 – -0.01 | **0.013** |
| Sex [male] * Time in KTx360° | 0.00 | -0.01 – 0.02 | 0.372 |
| Random Effects | | | |
| σ^2^ | 1.16 | | |
| τ_00_ _Code_eFA_ | 0.73 | | |
| ICC | 0.39 | | |
| N _Code_eFA_ | 725 | | |
| Observations | 2125 | | |

HADS = Hospital Anxiety and Depression Scale

reference categories: sex = female, first language = German, partnership = yes, donor type = living donation

Bold P-values indicate significant results (without Bonferroni correction).

Multilevel regression analyses for knowledge test and change in knowledge test (depicted as the interaction between time in KTx360° and sex). The interaction effect does not reach significance.

Supplementary Table 2. Age predicts the course of the knowledge level: total sample

| Predictors [category] | Estimates | CI | p |
| --- | --- | --- | --- |
| Intercept | 6.14 | 5.94 – 6.34 | **<0.001** |
| Age | -0.01 | -0.01 – 0.00 | 0.160 |
| Time in KTx360° | 0.02 | 0.02 – 0.03 | **<0.001** |
| Sex [male] | -0.24 | -0.40 – -0.07 | **0.005** |
| First language [non-German] | -0.72 | -0.99 – -0.45 | **<0.001** |
| Partnership [no] | -0.25 | -0.44 – -0.06 | **0.012** |
| Donor type [postmortal] | -0.12 | -0.31 – 0.06 | 0.198 |
| Time since KTx at enrollment into study | -0.02 | -0.03 – -0.00 | **0.018** |
| Years of education | 0.05 | 0.01 – 0.08 | **0.008** |
| Depression (HADS) | -0.03 | -0.05 – -0.01 | **0.013** |
| Age * Time in KTx360° | -0.00 | -0.00 – -0.00 | **0.006** |
| Random Effects | | | |
| σ^2^ | 1.15 | | |
| τ_00_ _Code_eFA_ | 0.74 | | |
| ICC | 0.39 | | |
| N _Code_eFA_ | 725 | | |
| Observations | 2125 | | |

HADS = Hospital Anxiety and Depression Scale

reference categories: sex = female, first language = German, partnership = yes, donor type = living donation

Bold P-values indicate significant results (without Bonferroni correction).

Supplementary Table 3. Age does not predict the course of the knowledge level: female subsample

| Predictors [category] | Estimates | CI | p |
| --- | --- | --- | --- |
| Intercept | 6.08 | 5.79 – 6.37 | **<0.001** |
| Age | -0.01 | -0.02 – 0.01 | 0.358 |
| Time in KTx360° | 0.02 | 0.01 – 0.03 | **<0.001** |
| First language [non-German] | -1.02 | -1.45 – -0.59 | **<0.001** |
| Partnership [no] | -0.10 | -0.39 – 0.19 | 0.492 |
| Donor type [postmortal] | -0.05 | -0.36 – 0.26 | 0.737 |
| Time since KTx at enrollment into study | -0.03 | -0.05 – -0.00 | **0.023** |
| Years of education | 0.01 | -0.05 – 0.07 | 0.737 |
| Depression (HADS) | 0.01 | -0.03 – 0.04 | 0.749 |
| Age * Time in KTx360° | -0.00 | -0.00 – 0.00 | 0.381 |
| Random Effects | | | |
| σ^2^ | 1.15 | | |
| τ_00_ _Code_eFA_ | 0.81 | | |
| ICC | 0.41 | | |
| N _Code_eFA_ | 299 | | |
| Observations | 879 | | |

HADS = Hospital Anxiety and Depression Scale

reference categories: first language = German, partnership = yes, donor type = living donation

Bold P-values indicate significant results (without Bonferroni correction).

Supplementary Table 4. Age predicts the course of the knowledge level: male subsample

| Predictors [category] | Estimates | CI | p |
| --- | --- | --- | --- |
| Intercept | 5.92 | 5.72 – 6.13 | **<0.001** |
| Age | -0.01 | -0.02 – 0.00 | 0.169 |
| Time in KTx360° | 0.02 | 0.02 – 0.03 | **<0.001** |
| First language [non-German] | -0.46 | -0.81 – -0.12 | **0.009** |
| Partnership [no] | -0.37 | -0.62 – -0.11 | **0.006** |
| Donor type [postmortal] | -0.15 | -0.38 – 0.08 | 0.195 |
| Time since KTx at enrollment into study | -0.01 | -0.03 – 0.00 | 0.146 |
| Years of education | 0.06 | 0.02 – 0.11 | **0.003** |
| Depression (HADS) | -0.04 | -0.07 – -0.02 | **0.001** |
| Age * Time in KTx360° | -0.00 | -0.00 – -0.00 | **0.004** |
| Random Effects | | | |
| σ^2^ | 1.15 | | |
| τ_00_ _Code_eFA_ | 0.67 | | |
| ICC | 0.37 | | |
| N _Code_eFA_ | 426 | | |
| Observations | 1246 | | |

HADS = Hospital Anxiety and Depression Scale

reference categories: first language = German, partnership = yes, donor type = living donation

Bold P-values indicate significant results (without Bonferroni correction).

Supplementary Table 5. Partnership does not predict the course of the knowledge level: total sample

| Predictors [category] | Estimates | CI | p |
| --- | --- | --- | --- |
| Intercept | 6.16 | 5.96 – 6.37 | **<0.001** |
| Partnership [no] | -0.34 | -0.56 – -0.12 | **0.003** |
| Time in KTx360° | 0.02 | 0.01 – 0.03 | **<0.001** |
| Sex [male] | -0.24 | -0.40 – -0.07 | **0.005** |
| First language [non-German] | -0.72 | -0.99 – -0.45 | **<0.001** |
| Donor type [postmortal] | -0.12 | -0.31 – 0.06 | 0.200 |
| Time since KTx at enrollment into study | -0.02 | -0.03 – -0.00 | **0.019** |
| Years of education | 0.05 | 0.01 – 0.08 | **0.008** |
| Age | -0.01 | -0.02 – -0.00 | **0.002** |
| Depression (HADS) | -0.03 | -0.05 – -0.01 | **0.013** |
| Partnership [no] * Time in KTx360° | 0.01 | -0.00 – 0.02 | 0.072 |
| Random Effects | | | |
| σ^2^ | 1.16 | | |
| τ_00_ _Code_eFA_ | 0.73 | | |
| ICC | 0.39 | | |
| N _Code_eFA_ | 725 | | |
| Observations | 2125 | | |

HADS = Hospital Anxiety and Depression Scale

reference categories: sex = female, first language = German, partnership = yes, donor type = living donation

Bold P-values indicate significant results (without Bonferroni correction).

Supplementary Table 6. Partnership does not predict the course of the knowledge level: female subsample

| Predictors [category] | Estimates | CI | p |
| --- | --- | --- | --- |
| Intercept | 6.06 | 5.76 – 6.35 | **<0.001** |
| Partnership [no] | -0.04 | -0.38 – 0.29 | 0.790 |
| Time in KTx360° | 0.02 | 0.01 – 0.03 | **<0.001** |
| First language [non-German] | -1.02 | -1.45 – -0.59 | **<0.001** |
| Donor type [postmortal] | -0.05 | -0.36 – 0.26 | 0.761 |
| Time since KTx at enrollment into study | -0.03 | -0.05 – -0.00 | **0.025** |
| Years of education | 0.01 | -0.05 – 0.07 | 0.721 |
| Age | -0.01 | -0.02 – 0.00 | 0.122 |
| Depression (HADS) | 0.01 | -0.03 – 0.04 | 0.770 |
| Partnership [no] * Time in KTx360° | -0.01 | -0.02 – 0.01 | 0.526 |
| Random Effects | | | |
| σ^2^ | 1.15 | | |
| τ_00_ _Code_eFA_ | 0.81 | | |
| ICC | 0.41 | | |
| N _Code_eFA_ | 299 | | |
| Observations | 879 | | |

HADS = Hospital Anxiety and Depression Scale

reference categories: sex = female, first language = German, partnership = yes, donor type = living donation

Bold P-values indicate significant results (without Bonferroni correction).

Supplementary Table 7. Partnership predicts the course of the knowledge level: male subsample

| Predictors [category] | Estimates | CI | p |
| --- | --- | --- | --- |
| Intercept | 5.99 | 5.78 – 6.20 | **<0.001** |
| Partnership [no] | -0.60 | -0.90 – -0.30 | **<0.001** |
| Time in KTx360° | 0.02 | 0.01 – 0.03 | **<0.001** |
| First language [non-German] | -0.45 | -0.80 – -0.11 | **0.010** |
| Donor type [postmortal] | -0.16 | -0.39 – 0.07 | 0.180 |
| Time since KTx at enrollment into study | -0.01 | -0.03 – 0.00 | 0.144 |
| Years of education | 0.06 | 0.02 – 0.10 | **0.004** |
| Age | -0.01 | -0.02 – -0.01 | **0.002** |
| Depression (HADS) | -0.04 | -0.07 – -0.02 | **0.001** |
| Partnership [no] * Time in KTx360° | 0.02 | 0.01 – 0.04 | **0.002** |
| Random Effects | | | |
| σ^2^ | 1.15 | | |
| τ_00_ _Code_eFA_ | 0.67 | | |
| ICC | 0.37 | | |
| N _Code_eFA_ | 426 | | |
| Observations | 1246 | | |

HADS = Hospital Anxiety and Depression Scale

reference categories: sex = female, first language = German, partnership = yes, donor type = living donation

Bold P-values indicate significant results (without Bonferroni correction).

Supplementary Table 8: Percentage of female and male participants answering the separate items of the knowledge test correctly.

|  | **Question 1** | | **Question 2** | | **Question 3** | | **Question 4** | | **Question 5** | | **Question 6** | | **Question 7** | | **Question 8** | |
| --- | --- | --- | --- | --- | --- | --- | --- | --- | --- | --- | --- | --- | --- | --- | --- | --- |
|  | female | male | female | male | female | male | female | male | female | male | female | male | female | male | female | male |
| **Baseline** | 88.1 | 74.9 | 94.4 | 91.2 | 55.2 | 55.6 | 80.3 | 81.3 | 59.6 | 52.3 | 77.7 | 80.6 | 69.0 | 63.5 | 61.8 | 62.6 |
| **3 months** | 91.2 | 89.4 | 91.2 | 95.5 | 70.2 | 66.7 | 73.7 | 72.7 | 61.4 | 47.0 | 80.7 | 84.8 | 73.7 | 77.3 | 71.9 | 83.3 |
| **6 months** | 89.5 | 83.9 | 95.2 | 92.5 | 60 | 64.6 | 78.1 | 78.9 | 60.0 | 57.8 | 84.8 | 81.4 | 70.5 | 75.2 | 69.5 | 75.8 |
| **9 months** | 94.9 | 85.1 | 95.0 | 93.1 | 56.7 | 65.5 | 81.7 | 81.6 | 63.3 | 52.9 | 86.7 | 77.0 | 88.3 | 72.4 | 73.3 | 74.7 |
| **12 months** | 90.4 | 87.8 | 94.1 | 90.0 | 67.6 | 63.0 | 76.5 | 82.9 | 66.9 | 56.1 | 86.0 | 88.3 | 73.5 | 74.4 | 64.7 | 72.2 |
| **18 months** | 88.9 | 85.3 | 94.4 | 88.7 | 64.8 | 65.6 | 83.3 | 81.5 | 72.2 | 59.6 | 89.8 | 88.7 | 79.6 | 79.5 | 73.1 | 68.9 |
| **24 months** | 91.3 | 90.2 | 95.7 | 91.0 | 63.0 | 69.2 | 80.4 | 74.4 | 64.1 | 54.1 | 85.9 | 88.0 | 83.7 | 79.7 | 70.7 | 76.7 |
| **30 months** | 91.1 | 91.8 | 97.8 | 91.8 | 75.6 | 67.2 | 71.1 | 78.7 | 57.8 | 52.5 | 91.1 | 93.4 | 82.2 | 80.3 | 82.2 | 77.4 |
| **36 months** | 94.1 | 91.3 | 100.0 | 95.7 | 70.6 | 65.2 | 64.7 | 73.9 | 58.8 | 65.2 | 100.0 | 91.3 | 94.1 | 91.3 | 82.4 | 87.0 |

Supplementary Table 9: Years of education do not predict the course of the knowledge level: total sample

| *Predictors [category]* | *Estimates* | *CI* | *p* |
| --- | --- | --- | --- |
| Intercept | 6.13 | 5.93 – 6.33 | **<0.001** |
| Years of education | 0.05 | 0.01 – 0.09 | **0.015** |
| Time in KTx360° | 0.02 | 0.02 – 0.03 | **<0.001** |
| Sex [male] | -0.24 | -0.40 – -0.07 | **0.005** |
| First language [non-German] | -0.73 | -0.99 – -0.46 | **<0.001** |
| Partnership [no] | -0.24 | -0.43 – -0.05 | **0.014** |
| Donor type [postmortal] | -0.12 | -0.31 – 0.06 | 0.195 |
| Time since KTx at enrollment into study | -0.02 | -0.03 – -0.00 | **0.021** |
| Age | -0.01 | -0.02 – -0.00 | **0.002** |
| Depression (HADS-D) | -0.03 | -0.05 – -0.01 | **0.012** |
| Years of education * Time in KTx360° | -0.00 | -0.00 – 0.00 | 0.708 |
| Random Effects | | | |
| σ^2^ | 1.16 | | |
| τ_00_ _Code_eFA_ | 0.73 | | |
| ICC | 0.39 | | |
| N _Code_eFA_ | 725 | | |
| Observations | 2125 | | |

HADS = Hospital Anxiety and Depression Scale

reference categories: sex = female, first language = German, partnership = yes, donor type = living donation

Bold P-values indicate significant results (without Bonferroni correction).

Supplementary Table 10: Years of education do not predict the course of the knowledge level: female subsample

| *Predictors [category]* | *Estimates* | *CI* | *p* |
| --- | --- | --- | --- |
| Intercept | 6.07 | 5.78 – 6.36 | **<0.001** |
| Years of education | 0.00 | -0.06 – 0.07 | 0.968 |
| Time in KTx360° | 0.02 | 0.01 – 0.03 | **<0.001** |
| First language [non-German] | -1.02 | -1.45 – -0.59 | **<0.001** |
| Partnership [no] | -0.10 | -0.39 – 0.19 | 0.506 |
| Donor type [postmortal] | -0.05 | -0.36 – 0.26 | 0.772 |
| Time since KTx at enrollment into study | -0.03 | -0.05 – -0.00 | **0.024** |
| Age | -0.01 | -0.02 – 0.00 | 0.128 |
| Depression (HADS-D) | 0.01 | -0.03 – 0.04 | 0.759 |
| Years of education * Time in KTx360° | 0.00 | -0.00 – 0.00 | 0.593 |
| Random Effects | | | |
| σ^2^ | 1.15 | | |
| τ_00_ _Code_eFA_ | 0.81 | | |
| ICC | 0.41 | | |
| N _Code_eFA_ | 299 | | |
| Observations | 879 | | |

HADS = Hospital Anxiety and Depression Scale

reference categories: sex = female, first language = German, partnership = yes, donor type = living donation

Bold P-values indicate significant results (without Bonferroni correction).

Supplementary Table 11: Years of education do not predict the course of the knowledge level: male subsample

| *Predictors [category]* | *Estimates* | *CI* | *p* |
| --- | --- | --- | --- |
| Intercept | 5.92 | 5.71 – 6.13 | **<0.001** |
| Years of education | 0.08 | 0.03 – 0.13 | **0.003** |
| Time in KTx360° | 0.02 | 0.02 – 0.03 | **<0.001** |
| First language [non-German] | -0.48 | -0.82 – -0.13 | **0.007** |
| Partnership [no] | -0.35 | -0.61 – -0.09 | **0.008** |
| Donor type [postmortal] | -0.16 | -0.39 – 0.07 | 0.168 |
| Time since KTx at enrollment into study | -0.01 | -0.03 – 0.01 | 0.167 |
| Age | -0.01 | -0.02 – -0.01 | **0.002** |
| Depression (HADS-D) | -0.04 | -0.07 – -0.02 | **0.001** |
| Years of education * Time in KTx360° | -0.00 | -0.00 – 0.00 | 0.309 |
| Random Effects | | | |
| σ^2^ | 1.16 | | |
| τ_00_ _Code_eFA_ | 0.66 | | |
| ICC | 0.36 | | |
| N _Code_eFA_ | 426 | | |
| Observations | 1246 | | |

HADS = Hospital Anxiety and Depression Scale

reference categories: sex = female, first language = German, partnership = yes, donor type = living donation

Bold P-values indicate significant results (without Bonferroni correction).

Supplementary Table 12: Depression (HADS-D) does not predict the course of the knowledge level: total sample

| *Predictors [category]* | *Estimates* | *CI* | *p* |
| --- | --- | --- | --- |
| Intercept | 6.13 | 5.93 – 6.33 | **<0.001** |
| Depression (HADS-D) | -0.03 | -0.05 – -0.00 | **0.043** |
| Time in KTx360° | 0.02 | 0.02 – 0.03 | **<0.001** |
| Sex [male] | -0.24 | -0.40 – -0.07 | **0.005** |
| First language [non-German] | -0.73 | -0.99 – -0.46 | **<0.001** |
| Partnership [no] | -0.24 | -0.43 – -0.05 | **0.014** |
| Donor type [postmortal] | -0.12 | -0.31 – 0.06 | 0.197 |
| Time since KTx at enrollment into study | -0.02 | -0.03 – -0.00 | **0.021** |
| Years of education | 0.05 | 0.01 – 0.08 | **0.008** |
| Age | -0.01 | -0.02 – -0.00 | **0.002** |
| Depression (HADS-D) * Time in KTx360° | -0.00 | -0.00 – 0.00 | 0.850 |
| Random Effects | | | |
| σ^2^ | 1.16 | | |
| τ_00_ _Code_eFA_ | 0.73 | | |
| ICC | 0.39 | | |
| N _Code_eFA_ | 725 | | |
| Observations | 2125 | | |

HADS = Hospital Anxiety and Depression Scale

reference categories: sex = female, first language = German, partnership = yes, donor type = living donation

Bold P-values indicate significant results (without Bonferroni correction).

Supplementary Table 13: Depression (HADS-D) does not predict the course of the knowledge level: female subsample

| *Predictors [category]* | *Estimates* | *CI* | *p* |
| --- | --- | --- | --- |
| Intercept | 6.08 | 5.79 – 6.37 | **<0.001** |
| Depression (HADS-D) | 0.02 | -0.02 – 0.06 | 0.383 |
| Time in KTx360° | 0.02 | 0.01 – 0.03 | **<0.001** |
| First language [non-German] | -1.02 | -1.45 – -0.59 | **<0.001** |
| Partnership [no] | -0.10 | -0.39 – 0.19 | 0.482 |
| Donor type [postmortal] | -0.05 | -0.36 – 0.26 | 0.747 |
| Time since KTx at enrollment into study | -0.03 | -0.05 – -0.00 | **0.025** |
| Years of education | 0.01 | -0.05 – 0.07 | 0.712 |
| Age | -0.01 | -0.02 – 0.00 | 0.125 |
| Depression (HADS-D) * Time in KTx360° | -0.00 | -0.00 – 0.00 | 0.243 |
| Random Effects | | | |
| σ^2^ | 1.15 | | |
| τ_00_ _Code_eFA_ | 0.81 | | |
| ICC | 0.41 | | |
| N _Code_eFA_ | 299 | | |
| Observations | 879 | | |

HADS = Hospital Anxiety and Depression Scale

reference categories: sex = female, first language = German, partnership = yes, donor type = living donation

Bold P-values indicate significant results (without Bonferroni correction).

Supplementary Table 14: Depression (HADS-D) does not predict the course of the knowledge level: male subsample

| *Predictors [category]* | *Estimates* | *CI* | *p* |
| --- | --- | --- | --- |
| Intercept | 5.92 | 5.71 – 6.13 | **<0.001** |
| Depression (HADS-D) | -0.05 | -0.08 – -0.02 | **0.002** |
| Time in KTx360° | 0.02 | 0.02 – 0.03 | **<0.001** |
| First language [non-German] | -0.47 | -0.82 – -0.13 | **0.008** |
| Partnership [no] | -0.35 | -0.61 – -0.10 | **0.007** |
| Donor type [postmortal] | -0.16 | -0.39 – 0.07 | 0.173 |
| Time since KTx at enrollment into study | -0.01 | -0.03 – 0.01 | 0.164 |
| Years of education | 0.06 | 0.02 – 0.10 | **0.004** |
| Age | -0.01 | -0.02 – -0.01 | **0.001** |
| Depression (HADS-D) * Time in KTx360 | 0.00 | -0.00 – 0.00 | 0.437 |
| Random Effects | | | |
| σ^2^ | 1.17 | | |
| τ_00_ _Code_eFA_ | 0.66 | | |
| ICC | 0.36 | | |
| N _Code_eFA_ | 426 | | |
| Observations | 1246 | | |

HADS = Hospital Anxiety and Depression Scale

reference categories: sex = female, first language = German, partnership = yes, donor type = living donation

Bold P-values indicate significant results (without Bonferroni correction).

Supplementary Table 15: First language does not predict the course of the knowledge level: total sample

| *Predictors [category]* | *Estimates* | *CI* | *p* |
| --- | --- | --- | --- |
| Intercept | 6.15 | 5.95 – 6.35 | **<0.001** |
| First language [non-German] | -0.90 | -1.22 – -0.58 | **<0.001** |
| Time in KTx360° | 0.02 | 0.02 – 0.03 | **<0.001** |
| Sex [male] | -0.24 | -0.40 – -0.07 | **0.005** |
| Partnership [no] | -0.24 | -0.43 – -0.05 | **0.015** |
| Donor type [postmortal] | -0.12 | -0.31 – 0.06 | 0.197 |
| Time since KTx at enrollment into study | -0.02 | -0.03 – -0.00 | **0.020** |
| Years of education | 0.05 | 0.01 – 0.08 | **0.008** |
| Age | -0.01 | -0.02 – -0.00 | **0.001** |
| Depression (HADS-D) | -0.03 | -0.05 – -0.01 | **0.013** |
| First language [non-German] * Time in KTx360° | 0.02 | -0.00 – 0.03 | 0.055 |
| Random Effects | | | |
| σ^2^ | 1.16 | | |
| τ_00_ _Code_eFA_ | 0.73 | | |
| ICC | 0.39 | | |
| N _Code_eFA_ | 725 | | |
| Observations | 2125 | | |

HADS = Hospital Anxiety and Depression Scale

reference categories: sex = female, first language = German, partnership = yes, donor type = living donation

Bold P-values indicate significant results (without Bonferroni correction).

Supplementary Table 16: First language does not predict the course of the knowledge level: female subsample

| *Predictors [category]* | *Estimates* | *CI* | *p* |
| --- | --- | --- | --- |
| Intercept | 6.11 | 5.82 – 6.40 | **<0.001** |
| First language [non-German] | -1.27 | -1.77 – -0.76 | **<0.001** |
| Time in KTx360° | 0.02 | 0.01 – 0.03 | **<0.001** |
| Partnership [no] | -0.10 | -0.39 – 0.19 | 0.501 |
| Donor type [postmortal] | -0.06 | -0.37 – 0.25 | 0.716 |
| Time since KTx at enrollment into study | -0.03 | -0.05 – -0.00 | **0.024** |
| Years of education | 0.01 | -0.05 – 0.07 | 0.734 |
| Age | -0.01 | -0.02 – 0.00 | 0.128 |
| Depression (HADS-D) | 0.01 | -0.03 – 0.04 | 0.762 |
| First language [non-German] * Time in KTx360° | 0.02 | -0.00 – 0.05 | 0.064 |
| Random Effects | | | |
| σ^2^ | 1.14 | | |
| τ_00_ _Code_eFA_ | 0.81 | | |
| ICC | 0.41 | | |
| N _Code_eFA_ | 299 | | |
| Observations | 879 | | |

HADS = Hospital Anxiety and Depression Scale

reference categories: sex = female, first language = German, partnership = yes, donor type = living donation

Bold P-values indicate significant results (without Bonferroni correction).

Supplementary Table 17: First language does not predict the course of the knowledge level: male subsample

| *Predictors [category]* | *Estimates* | *CI* | *p* |
| --- | --- | --- | --- |
| Intercept | 5.93 | 5.72 – 6.14 | **<0.001** |
| First language [non-German] | -0.58 | -1.00 – -0.16 | **0.007** |
| Time in KTx360° | 0.02 | 0.02 – 0.03 | **<0.001** |
| Partnership [no] | -0.35 | -0.61 – -0.09 | **0.008** |
| Donor type [postmortal] | -0.16 | -0.39 – 0.07 | 0.178 |
| Time since KTx at enrollment into study | -0.01 | -0.03 – 0.01 | 0.159 |
| Years of education | 0.06 | 0.02 – 0.10 | **0.004** |
| Age | -0.01 | -0.02 – -0.01 | **0.001** |
| Depression (HADS-D) | -0.04 | -0.07 – -0.02 | **0.001** |
| First language [non-German] * Time in KTx360° | 0.01 | -0.01 – 0.03 | 0.378 |
| Random Effects | | | |
| σ^2^ | 1.17 | | |
| τ_00_ _Code_eFA_ | 0.66 | | |
| ICC | 0.36 | | |
| N _Code_eFA_ | 426 | | |
| Observations | 1246 | | |

HADS = Hospital Anxiety and Depression Scale

reference categories: sex = female, first language = German, partnership = yes, donor type = living donation

Bold P-values indicate significant results (without Bonferroni correction).

Supplementary Table 18: Time since KTx at enrollment into study does not predict the course of the knowledge level: total sample

| *Predictors [category]* | *Estimates* | *CI* | *p* |
| --- | --- | --- | --- |
| Intercept | 6.13 | 5.93 – 6.33 | **<0.001** |
| Time since KTx at enrollment into study | -0.01 | -0.03 – 0.01 | 0.215 |
| Time in KTx360° | 0.02 | 0.02 – 0.03 | **<0.001** |
| First language [non-German] | -0.72 | -0.99 – -0.46 | **<0.001** |
| Sex [male] | -0.24 | -0.40 – -0.07 | **0.005** |
| Partnership [no] | -0.24 | -0.43 – -0.05 | **0.015** |
| Donor type [postmortal] | -0.12 | -0.31 – 0.06 | 0.198 |
| Years of education | 0.05 | 0.01 – 0.08 | **0.008** |
| Age | -0.01 | -0.02 – -0.00 | **0.001** |
| Depression (HADS-D) | -0.03 | -0.05 – -0.01 | **0.012** |
| Time since KTx at enrollment into study * Time in KTx360° | -0.00 | -0.00 – 0.00 | 0.292 |
| Random Effects | | | |
| σ^2^ | 1.16 | | |
| τ_00_ _Code_eFA_ | 0.73 | | |
| ICC | 0.39 | | |
| N _Code_eFA_ | 725 | | |
| Observations | 2125 | | |

HADS = Hospital Anxiety and Depression Scale

reference categories: sex = female, first language = German, partnership = yes, donor type = living donation

Bold P-values indicate significant results (without Bonferroni correction).

Supplementary Table 19: Time since KTx at enrollment into study does not predict the course of the knowledge level: female subsample

| *Predictors [category]* | *Estimates* | *CI* | *p* |
| --- | --- | --- | --- |
| Intercept | 6.08 | 5.79 – 6.37 | **<0.001** |
| Time since KTx at enrollment into study | -0.02 | -0.05 – 0.01 | 0.186 |
| Time in KTx360° | 0.02 | 0.01 – 0.03 | **<0.001** |
| First language [non-German] | -1.02 | -1.45 – -0.59 | **<0.001** |
| Partnership [no] | -0.10 | -0.39 – 0.19 | 0.503 |
| Donor type [postmortal] | -0.05 | -0.36 – 0.26 | 0.747 |
| Years of education | 0.01 | -0.05 – 0.07 | 0.715 |
| Age | -0.01 | -0.02 – 0.00 | 0.120 |
| Depression (HADS-D) | 0.01 | -0.03 – 0.04 | 0.756 |
| Time since KTx at enrollment into study * Time in KTx360° | -0.00 | -0.00 – 0.00 | 0.392 |
| Random Effects | | | |
| σ^2^ | 1.15 | | |
| τ_00_ _Code_eFA_ | 0.81 | | |
| ICC | 0.41 | | |
| N _Code_eFA_ | 299 | | |
| Observations | 879 | | |

HADS = Hospital Anxiety and Depression Scale

reference categories: sex = female, first language = German, partnership = yes, donor type = living donation

Bold P-values indicate significant results (without Bonferroni correction).

Supplementary Table 20: Time since KTx at enrollment into study does not predict the course of the knowledge level: male subsample

| *Predictors [category]* | *Estimates* | *CI* | *p* |
| --- | --- | --- | --- |
| Intercept | 5.92 | 5.71 – 6.13 | **<0.001** |
| Time since KTx at enrollment into study | -0.01 | -0.03 – 0.01 | 0.476 |
| Time in KTx360° | 0.02 | 0.02 – 0.03 | **<0.001** |
| First language [non-German] | -0.47 | -0.82 – -0.13 | **0.007** |
| Partnership [no] | -0.35 | -0.61 – -0.09 | **0.007** |
| Donor type [postmortal] | -0.16 | -0.39 – 0.07 | 0.176 |
| Years of education | 0.06 | 0.02 – 0.10 | **0.004** |
| Age | -0.01 | -0.02 – -0.01 | **0.001** |
| Depression (HADS-D) | -0.04 | -0.07 – -0.02 | **0.001** |
| Time since KTx at enrollment into study * Time in KTx360° | -0.00 | -0.00 – 0.00 | 0.486 |
| Random Effects | | | |
| σ^2^ | 1.17 | | |
| τ_00_ _Code_eFA_ | 0.66 | | |
| ICC | 0.36 | | |
| N _Code_eFA_ | 426 | | |
| Observations | 1246 | | |

HADS = Hospital Anxiety and Depression Scale

reference categories: sex = female, first language = German, partnership = yes, donor type = living donation

Bold P-values indicate significant results (without Bonferroni correction).

Supplementary Table 21: Donor type does not predict the course of the knowledge level: total sample

| *Predictors [category]* | *Estimates* | *CI* | *p* |
| --- | --- | --- | --- |
| Intercept | 6.13 | 5.91 – 6.34 | **<0.001** |
| Donor type [postmortal] | -0.11 | -0.33 – 0.10 | 0.299 |
| Time in KTx360° | 0.02 | 0.01 – 0.03 | **<0.001** |
| Sex [male] | -0.24 | -0.40 – -0.07 | **0.005** |
| First language [non-German] | -0.73 | -0.99 – -0.46 | **<0.001** |
| Partnership [no] | -0.24 | -0.43 – -0.05 | **0.014** |
| Time since KTx at enrollment into study | -0.02 | -0.03 – -0.00 | **0.021** |
| Years of education | 0.05 | 0.01 – 0.08 | **0.008** |
| Age | -0.01 | -0.02 – -0.00 | **0.002** |
| Depression (HADS-D) | -0.03 | -0.05 – -0.01 | **0.012** |
| Donor type [postmortal] * Time in KTx360° | -0.00 | -0.01 – 0.01 | 0.908 |
| Random Effects | | | |
| σ^2^ | 1.16 | | |
| τ_00_ _Code_eFA_ | 0.73 | | |
| ICC | 0.39 | | |
| N _Code_eFA_ | 725 | | |
| Observations | 2125 | | |

HADS = Hospital Anxiety and Depression Scale

reference categories: sex = female, first language = German, partnership = yes, donor type = living donation

Bold P-values indicate significant results (without Bonferroni correction).

Supplementary Table 22: Donor type does not predict the course of the knowledge level: female subsample

| *Predictors [category]* | *Estimates* | *CI* | *p* |
| --- | --- | --- | --- |
| Intercept | 6.09 | 5.77 – 6.41 | **<0.001** |
| Donor type [postmortal] | -0.07 | -0.43 – 0.29 | 0.716 |
| Time in KTx360° | 0.02 | 0.00 – 0.03 | **0.013** |
| First language [non-German] | -1.02 | -1.45 – -0.59 | **<0.001** |
| Partnership [no] | -0.10 | -0.39 – 0.19 | 0.507 |
| Time since KTx at enrollment into study | -0.03 | -0.05 – -0.00 | **0.025** |
| Years of education | 0.01 | -0.05 – 0.07 | 0.718 |
| Age | -0.01 | -0.02 – 0.00 | 0.127 |
| Depression (HADS-D) | 0.01 | -0.03 – 0.04 | 0.753 |
| Donor type [postmortal] * Time in KTx360° | 0.00 | -0.02 – 0.02 | 0.840 |
| Random Effects | | | |
| σ^2^ | 1.15 | | |
| τ_00_ _Code_eFA_ | 0.81 | | |
| ICC | 0.41 | | |
| N _Code_eFA_ | 299 | | |
| Observations | 879 | | |

HADS = Hospital Anxiety and Depression Scale

reference categories: sex = female, first language = German, partnership = yes, donor type = living donation

Bold P-values indicate significant results (without Bonferroni correction).

Supplementary Table 23: Donor type does not predict the course of the knowledge level: male subsample

| *Predictors [category]* | *Estimates* | *CI* | *p* |
| --- | --- | --- | --- |
| Intercept | 5.91 | 5.68 – 6.14 | **<0.001** |
| Donor type [postmortal] | -0.14 | -0.42 – 0.13 | 0.297 |
| Time in KTx360° | 0.03 | 0.01 – 0.04 | **<0.001** |
| First language [non-German] | -0.48 | -0.82 – -0.13 | **0.007** |
| Partnership [no] | -0.35 | -0.61 – -0.10 | **0.007** |
| Time since KTx at enrollment into study | -0.01 | -0.03 – 0.01 | 0.161 |
| Years of education | 0.06 | 0.02 – 0.10 | **0.004** |
| Age | -0.01 | -0.02 – -0.01 | **0.001** |
| Depression (HADS-D) | -0.04 | -0.07 – -0.02 | **0.001** |
| Donor type [postmortal] * Time in KTx360° | -0.00 | -0.02 – 0.01 | 0.841 |
| Random Effects | | | |
| σ^2^ | 1.17 | | |
| τ_00_ _Code_eFA_ | 0.66 | | |
| ICC | 0.36 | | |
| N _Code_eFA_ | 426 | | |
| Observations | 1246 | | |

HADS = Hospital Anxiety and Depression Scale

reference categories: sex = female, first language = German, partnership = yes, donor type = living donation

Bold P-values indicate significant results (without Bonferroni correction).
